# Proteomics Is Analytical Chemistry: Fitness-for-Purpose in the Application of Top-Down and Bottom-Up Analyses

**DOI:** 10.3390/proteomes3040440

**Published:** 2015-12-03

**Authors:** Jens R. Coorssen, Alfred L. Yergey

**Affiliations:** 1 Molecular Physiology and the Molecular Medicine Research Group, School of Medicine, Western Sydney University, Office 30.2.15, Campbelltown Campus, Locked Bag 1797, Penrith 2570, NSW, Australia; 2 National Institute of Child Health and Human Development, NIH, Building 10, Room 9D 52, Bethesda, MD 20892, USA

**Keywords:** 2DE, Bottom-up, Discovery proteomics, LC/MS/MS, protein species, proteoforms, shotgun, Top-down, two-dimensional gel electrophoresis

## Abstract

Molecular mechanisms underlying health and disease function at least in part based on the flexibility and fine-tuning afforded by protein isoforms and post-translational modifications. The ability to effectively and consistently resolve these protein species or proteoforms, as well as assess quantitative changes is therefore central to proteomic analyses. Here we discuss the pros and cons of currently available and developing analytical techniques from the perspective of the full spectrum of available tools and their current applications, emphasizing the concept of fitness-for-purpose in experimental design based on consideration of sample size and complexity; this necessarily also addresses analytical reproducibility and its variance. Data quality is considered the primary criterion, and we thus emphasize that the standards of Analytical Chemistry must apply throughout any proteomic analysis.

“He who acknowledges the imperfections of his instrument, and makes allowance for it in discussing his observations, is in a much better position for gaining truth than if he claimed his instrument to be infallible...”

William James [1]

## 1. Introduction

In the current post-genomic era of large scale ‘omic analyses, Proteomics occupies a central position due to the vast diversity of functional and structural roles for proteins. To understand both normal and dysfunctional physiological states necessitates a quantitative understanding of protein alterations, both across the proteome as well as to individual proteins. Indeed, in addition to the breadth of functional and regulatory capacity introduced by splice variants and isoforms, the quantity, location, and functional states of proteins are continuously fine-tuned by myriad potential post-translational modifications (PTM). Understanding variations in both quantity and protein species or proteoforms, is thus central to understanding much of biology. Accordingly, we use the terms proteoform and protein species interchangeably here in an effort to avoid any semantic ambiguities [2,3,4].

The question then is how to address the issue of characterizing these species. In the first instance one might consider generating a set of “protein identifications.” In reality, this has often been simply “proteins detected” and has been the widely accepted approach of the past decade. This must be recognized, however, as little more than an exercise in cataloging without specification. To use an analogy with taxonomy, it is as if one were to say genus *Homo* without specifying *sapiens* or *erectus*.

Here we discuss the critical concept that to effectively dissect and understand molecular mechanisms that underlie cellular functions requires the use of ***Top-down*** proteome analyses that resolve the full complement of proteoforms present. This does not deny the value and even the importance of detecting a large number of proteins, but at the same time it requires the recognition that such cataloging or detection is not a complete specification (*i.e.*, “species-fication”) regardless of the approach used to assess a proteome.

Going beyond protein cataloging or specification to a detailed quantification of particular proteoforms adds an immense additional layer of complexity to the overall problem. All three of these tasks—cataloging, specification and quantification—offer particular and useful insights to various issues across the breadth of biological research. Over the last 25 years, all three of these tasks have been undertaken in one form or another by use of a variety of methods most of which employ mass spectrometry (MS) to a greater or lesser extent.

Proteomics thus offers critical insights over the full spectrum of biological and biomedical research. Classical protein analyses required at least micromolar amounts of highly purified proteins; use of MS and refined protein detection methods [5,6,7,8,9] have circumvented this limitation and expanded the capabilities to analyze complex mixtures of proteins and other molecules. Despite the broad applicability of MS approaches, as with any method, there must nonetheless also be a clear and realistic recognition of their general as well as specific limitations. More recently, a number of approaches to the problems of analysis and determination have been developed but like many changes to proteome analysis, most have yet to be critically examined, in parallel, across a large number of research sites. When this has been attempted, even with some regularity by the three different Proteomics Research Groups within the Association of Biomolecular Research Facilities, the irreproducibility and often poor quality of the resulting data from site-to-site should raise serious concerns [10,11]. This is further emphasized by recent and critical calls for better standardization of methods and analyses [12,13,14]. It must also be recognized that while a select few groups have regular and continuing access to the newest and even prototype LC and MS instrumentation, the bulk of the Proteomics done internationally must contend with and thus excel as best as possible with largely robust and proven instruments, but not the “latest and greatest”. Thus, while instrument development is clearly a critical part of Proteomics, as any research discipline, one must critically consider how broadly applicable and reproducible are the processes, protocols, and resulting data across both research teams as well as sample types.

## 2. Fitness-for-Purpose

A trend in the field has been to define approaches for assessing the proteome based on the specific instrumentation adopted; here we propose that other criteria—specifically the underlying features or elements of the strategy—better distinguish between and define these approaches. Further, we suggest that the different strategies that are available offer competitive strengths and weaknesses—no single approach is optimal under all circumstances; in this regard they might best be considered complementary. Rather than simply throwing a given technology at a sample and assuming it sufficient, we suggest that it is critical to consider the relative merits of each strategy. In particular, we suggest that the concept of *fitness-for-purpose*, as commonly adopted in other areas of Analytical Chemistry, should be used to define the analytical objectives based on both the nature of the sample and the goal(s) of the analyses. Unfortunately, while this is the ideal, the combination of available instrumentation for any research group might not be the best match for the problem being investigated.

The issue is further complicated depending upon the nature of the analysis undertaken. We suggest that there is a three-fold hierarchy to the tasks mentioned above and that in order of increasing difficulty they are (1) catalog as many proteins as possible in a given sample; (2) specify protein alterations of unknown nature and magnitude; and, finally, (3) develop a list of “targets” for (absolute) quantification (see below). This latter is possibly the ultimate goal of all efforts in Proteomics and requires validation of target species, and the development of standards and meaningful linear response ranges. The analytical approaches required for each of these tasks, while often closely related, differ sufficiently that the argument of fitness-for-purpose seems quite appropriate.

**Figure 1:**
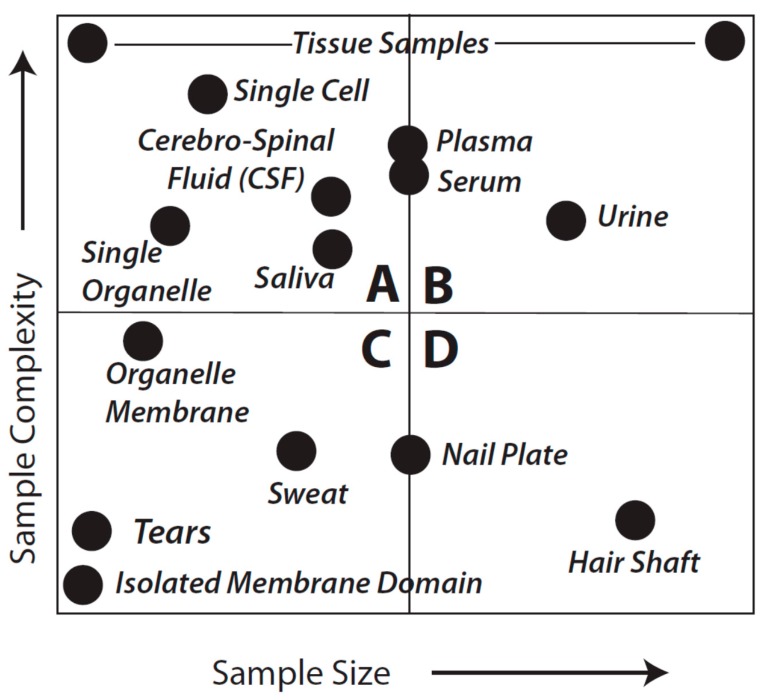
Idealized sample types characterized by complexity and amount of material. This is not meant to be an exact reflection of each and every sample type, known or unknown, or even to imply that we appreciate the complexity of every sample. Quadrants are arbitrarily identified as **A**–**D** and discussed in detail in the text.

Here we discuss the pros and cons of currently available and developing analytical techniques from the perspective of the full spectrum of available tools and their current applications. The use of fitness-for-purpose in the context of experimental design includes not only instrumentation available for the measurements, but also sample size and complexity; it also incorporates the manner in which analytical reproducibility and its variance are considered. Currently, Proteomics practitioners use a diversity of sample preparation and analytical tools to assess a wide range of sample types that can vary in complexity and quantity of material available. Figure 1 provides a general overview that aims to facilitate further consideration of sample types and analytical objectives. As might be expected, the considerations of fitness-for-purpose vary with each of the quadrants shown in the Figure.

## 3. Analytical Options *vs.* Goals

The selection of the analytical option (*i.e.*, broadly, Top-down or Bottom-up; see below) to be used in each quadrant is complicated further by the specific goals. That is, the option for class (1), cataloging as many proteofroms as possible within a given sample including resolving and characterizing all isoforms, splice variants, and their PTM (e.g., phosphorylation, glycosylation, oxidation, palmitoylation, and so forth) differs from the options for class (2), identifying specific mutations of a single protein (e.g., hemoglobin) and differs again for problems in class (3), quantifying a single protein in a complex mixture in which the analyte of interest may be a very minor component within an extremely wide dynamic range of concentrations in which sensitivity of detection and linear dynamic range issues arise. The considerations of fitness-for-purpose vary appreciably for each of these.

Unfortunately, there is a limited universe of strategies available to address the complexity of proteomes—in large part because of PTM, isoforms, mutations and/or variants (*i.e.*, the full diversity of proteoforms) as well as the exceedingly broad dynamic range of analyte quantities. When these considerations are added to the reality of the diverse nature of samples delineated in Figure 1, there is clear need for thoughtful consideration of how these stratagems should be applied in terms of fitness-for-purpose. It is these considerations that are covered in the balance of this perspective.

Currently, there are two concepts that define the approaches to proteome analysis: Top-down and Bottom-up Proteomics. The Top-down concept has been used in Proteomics and its predecessor, protein (bio)chemistry, since the development of two-dimensional gel electrophoresis (2DE) and perhaps even earlier with the application of antecedent electrophoretic approaches. Top-down approaches thus also include any other strategies that preserve intact protein species prior to MS analyses, including other electrophoretic and chromatographic methods that inform about physiochemical properties (other than apparent molecular weight or isoelectric point, e.g., hydrophobicity). For the last two decades proteins resolved by 2DE have been identified using MS-based analyses, the potential of this combined 2DE/MS approach being first recognized in the mid-1990s [15,16]. While still requiring extensive up-front protein resolution using multiple approaches, more recent MS-intensive top-down approaches have been refined to the point that substantial sequence coverage of a given purified protein is achievable [17,18]. This approach continues to rapidly evolve and, while it has immense promise and showcases developing MS instrumentation, it is far from routine and may in fact not prove routinely affordable or fully applicable for quite some time; it may also remain somewhat more challenging than gel-based methods since the peptides assessed after the resolution of protein species by 2DE are immensely easier to deal with than intact proteins (*i.e.*, for reasons of size, limited folding, and a narrower range of hydrophobicity). Nonetheless, it is clear that evolving technology will directly address these issues in the coming decade(s) [17,18,19,20,21,22].

In all cases, the Top-down approach involves the large-scale (quantitative) resolution of “all the proteins” extracted from a given biological sample, with the implicit goal of retaining as much information as possible about each species (*i.e.*, pI, MW, PTM, isoforms, quantity, and so forth). Thus, the most logical application of the term Top-down, with reference to the fullest range of analytical Proteomic strategies being used internationally, still refers to those analyses moving from the resolution of intact proteoforms (thereby preserving other critical information) to sequence and structural analyses. Thus, by definition, Top-down does not merely mean introducing intact proteins into a mass spectrometer, despite the opinion of some members of the MS community, but most certainly includes intact proteoforms resolved by any method (e.g., 2DE—electrochromatography in a gel matrix prior to MS analysis). Top-down reflects the nature of the analysis (the analytical pathway) and should not be confused with some current instrumentation-centric uses of the term [23].

The term Bottom-up is a more recent development, denoting a collection of methods broadly referred to as “shotgun” approaches (e.g., originating with MudPit [24]) in which a mixture of proteins (*i.e.*, a tissue extract) is digested reproducibly; essentially a complex mixture of proteins is cleaved to yield a substantially even more complex mixture of peptides that are then fractionated using multi-step LC, and these are then introduced into a mass spectrometer. The Bottom-up terminology is therefore instrumentation-centric and refers specifically to the reconstruction of protein sequences based on the determination of some number of individual peptides from the fractionated complex digest mixture. It should be noted that subsequent to the digestion, information concerning many physical-chemical characteristics of the intact proteins is lost, particularly pI and molecular weight, making subsequent delineation of functionally-relevant species somewhat inferential. Furthermore, while some random number of modified peptides can sometimes be identified in standard shotgun processes, there is little if any evidence of routine or standardized identifications of any PTM without selectively targeted approaches (see below). Nevertheless, because of the supposed ease of such analyses these approaches have become quite popular. However, the same considerations of sample complexity and variance apply to either Top-down or Bottom-up approaches. 

Good analytical practice would require the minimal use of at least three technical replicates per sample regardless of the technique used. 2DE-based Top-down approaches address analytical variance by employing the *parallel* resolution of multiple aliquots of the same sample (*i.e.*, a minimum of three replicate gels resolved in parallel per sample). Bottom-up approaches also require that at least triplicate aliquots of the fractionated peptides of each digested sample be analyzed in order to achieve the same level of reproducibility. By necessity this requires *sequential* analytical runs rather than parallel ones (*i.e.*, three strong cation fractionations per LC/MS/MS run per experimental sample) [25]. In addition, the development and broader application of statistical tools for assigning significance to peptide “identifications” has introduced somewhat more systematic rigor into the process of using information available in databases. As key experts have noted, the False Discovery Rate (FDR) and the Posterior Error Probability (PEP) scores are complementary but how they are used depends on the nature of subsequent experiments or conclusions being drawn from the data, and that they are best applied to protein identifications rather than just to peptides *per se* [26,27]. We note also that the quality of available databases (*i.e.*, at the time of interrogation) as well as the nature of decoy databases play heavily on the quality of the resulting FDR and PEP scores. An additional, more recently identified analytical issue has to do with the long-held and widespread assumption that each MS/MS spectrum corresponds to only a single peptide. It is now recognized that chimeric MS/MS spectra arising from co-eluting and co-fragmenting peptides can increase the FDR in subsequent database searches. Newer instrumentation and analytical approaches appear to enable marked improvements in the deconvolution of spectra and thus peptide identifications [28,29,30].

## 4. Fitness-for-Purpose and the Three Analytical Goals

**Figure 2:**
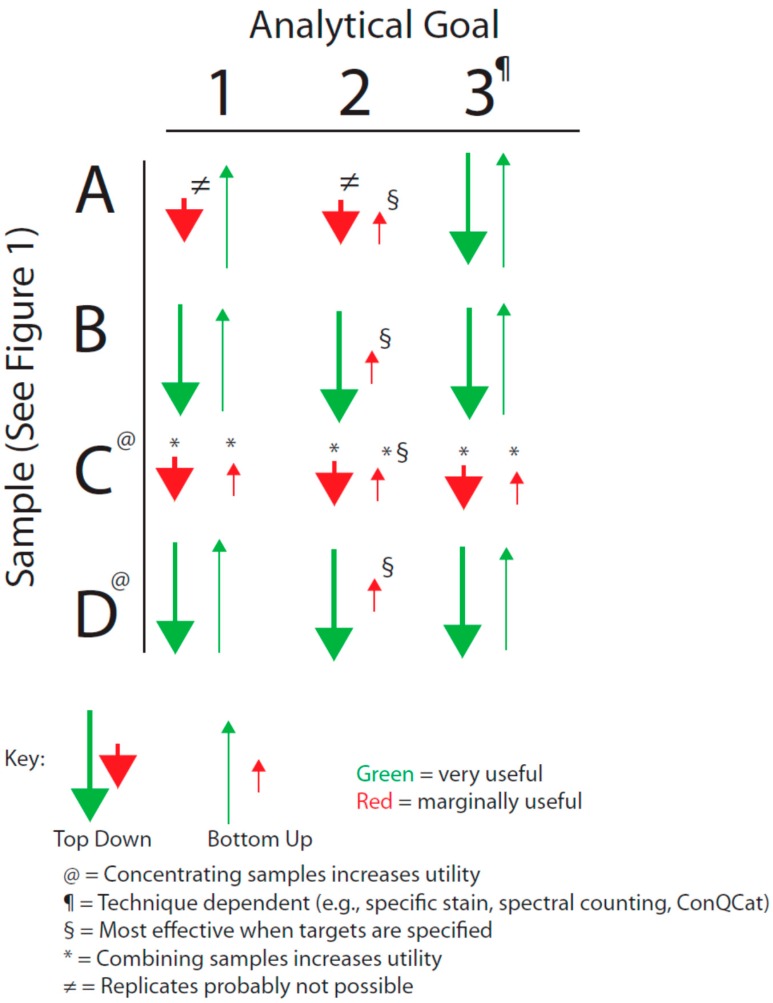
Decision matrix based on sample type and analytical goal (see text). **A**, **B**, **C**, and **D** correspond to the quadrants identified in Figure 1.

We have sought to visualize the issues raised by the general breadth of sample types shown in Figure 1 with the analytical goals mentioned above by forming an analytical decision matrix that is outlined in Figure 2. This matrix must also be considered in the light of the issues of analytical variance just mentioned.

### 4.1. Analytical Goal 1: Cataloging as Many Protein Species as Possible

Quadrants B and D of Figure 1 are equally amenable to application of either Top-down or Bottom-up approaches; thus, *in theory* they are equally fit to the purpose of cataloging protein species although almost certainly vary in quantitative rigor, particularly in terms of the practicality and critical importance of addressing the likely range of proteoforms by using a Bottom-up approach (see below). For samples that are in the class characterized by Quadrant A, shotgun methods may sometimes be preferable; such samples might include a CSF sample from a baby, a fine needle biopsy from a single patient, or even single cells or single organelles. While certainly capable of resolving such samples, it seems less likely that Top-down methods could currently be used for multiple replicates of individual samples in this quadrant (although, again, techniques and technology are evolving). However, use of pooled samples could circumvent this issue although individual subject information would be lost along with a sense of biological variability. The latter is likely less of an issue when trying to first identify critical protein alterations, for instance in a given disease state relative to healthy controls. Nevertheless, perhaps the most difficult analyses are characterized by those samples found in Quadrant C. In the extreme, such samples would include tears or even an isolated membrane domain or protein complex. Shotgun methods may yield some results but more than likely only for hyper-abundant proteins and with “identifications” based on very low sequence coverage.

Samples defined by Quadrants A and C thus raise a critical point: no matter which of the technical approaches is employed an overriding consideration of any result is quality. In order to adequately identify a protein we argue that an absolute minimum of three peptides is required; we propose that a minimum 8%–10% coverage across the *entire* sequence is required to comfortably identify a protein based on current databases. If one were to detect three peptides or 30% of a total sequence, and all three were in the amino terminal region of the protein, the question must be raised about the validity of identifying that protein—assuredly a portion or processed version of the protein is likely present, but not the full protein/proteoform. As we have seen on numerous occasions in our gel-based Top-down studies, we detect the “same” protein in completely different regions of the gel—a consequence of speciation by any number of proteolytic or other PTM. Furthermore, with regard to analysis, the size of the protein is largely irrelevant if it has sufficient cleavage sites for the protease(s) being used and the resolving system is up to the task; just another reason that there tends to be substantially better coverage and thus identifications using Top-down proteomic approaches that first isolate intact proteoforms. With the limited sequence coverage that often arises from Bottom-up approaches also comes the need to validate protein identifications using alternate means (e.g., immunoblotting), although this can rarely if ever provide information concerning specific proteoforms. Nonetheless, such validation should be the norm, and expected of any protein identifications based on extremely limited sequence coverage. Notable as well is the importance of critically considering which protease to use during the analytical process so as to ensure detection of specific PTM [31].

However, no matter what the sequence length, certainly more than a single peptide is required for any realistic and confident identification of a proteoform. An unfortunate and problematic aspect of the Bottom-up methods is that, even ignoring the loss of pI information, with the loss of MW information it is virtually impossible to distinguish a truncated species from one that is full length unless the peptides found provide coverage ranging over the entire proteoform sequence and may nonetheless still indicate the potential presence of more than one species depending on the quality of digestion and peptide recovery. Nevertheless, these methods remain fit for the purpose of broadly cataloging *potential* proteins in the sample. Of course, in order to reliably detect PTM, isoforms or splice variants, a much greater fraction of the total sequence must be obtained; alternatively, prefractionation or enrichment protocols are typically applied, requiring completely separate analyses (with full technical replicates) to effectively identify each and every different potential modification. In contrast, these physiologically important alternate or modified species are all well-resolved, in parallel, in the same analytical process when using 2DE (of late also coupled with postfractionation and deep imaging to yield better resolution and detection of protein species and thus maximal data per gel) [9,10,32]. In terms of technical replicates and in-depth peptide analyses, there seems little question from a scientific standpoint that rate of throughput is still unlikely to be a major selling point for either approach to proteome analyses if data quality remains the final critical evaluation (and therefore perhaps throughput is something that should be far less dwelled upon). Thus, perhaps quality of the data should remain the final genuine point of importance in any ‘omics analysis?

### 4.2. Analytical Goal 2: Finding a Protein with a Specific Mutation

This is an appreciably more challenging problem than Analytical Goal 1 since it requires, at a minimum, isolating or resolving a specific proteoform or, if the mutation is known, perhaps even a specific peptide in that species. This problem is further compounded if the protein of interest is not very abundant. It is generally held that, ideally, such work should involve some preliminary sample preparation to reduce the complexity of any mixture of protein species. Commonly, techniques involving affinity isolation are used, raising the cautionary concern of potential losses of the analyte(s) of interest (*i.e.*, before or during elution after the isolation step). If this is a targeted approach (e.g., the mutation is known) then either Bottom-up or Top-down strategies may be equally fit for the purpose since the former can be made to focus on specific peptides (and thus, theoretically, even proteoforms). If however the mutation is not well characterized, the Top-down approach is likely to be more fit for the purpose in that proteoforms are first isolated (*i.e.*, purified) thereby enabling more extensive coverage in subsequent MS analyses. Once again, samples existing in Quadrants A and C of Figure 1 remain problematic.

### 4.3. Analytical Goal 3: Quantifying a Single Protein in a Complex Mixture

The general problem of protein quantification in complex mixtures remains quite challenging [33]. There are a number of widely employed methods that more often than not provide relative quantification even between many proteoforms although the quality of several such analyses remain appropriately debated in the field. Absolute (*i.e.*, molecular-level) quantification, which is the ultimate aim of this objective from a strictly analytical perspective, requires use of one or more internal standards, thereby making it a targeted approach that is only applicable when the specific protein species of interest is already known (e.g., QConCat, SISCAPA, SRM/MRM, and MALDI) [33,34,35]. As such techniques are essentially instrument-dependent, either Top-down or Bottom-up approaches are equally fit-for-purpose. In general, however, the best results, in terms of minimized variance, of any mass spectrometric-based quantification are obtained when the analytes are relatively clean and thus as free as possible of interferences. As discussed in Objective 2, appropriate sample cleanup methods are likely to be necessary when doing such quantitative analyses. Notably, absolute quantification requires use not only of appropriate standards, but a calibration curve of said standards over a range of concentrations consistent with changes in the amount of analyte. The calibration curves are established based on the ratio of the responses of the analyte *vs.* the calibration standards (*i.e.*, the response ratio).

A further issue for consideration is that an absolute quantification reflect as much as possible the specific molecular entity being quantified. Therefore, for a protein (or more specifically a given proteoform), this again raises the necessity for breadth of coverage of the analyte. Current methods tend to focus on only a single peptide based on the concept of a so-called “proteotypic” sequence. Such notions of “unique” peptides are completely database-dependent and do not take into consideration our incomplete knowledge of the full complement of proteins let alone proteoforms that may arise from a given genome and physiological state. Accordingly, at least two *or more* standard or calibration peptides per protein, corresponding to quite spatially distinct regions spanning the full primary sequence (and any specifically post-translationally modified sequences), are required to ensure effective quantification of a given species. The results of the response ratios of all calibration peptides must be incorporated into the final quantification; lack of internal consistency of the response ratios would tend to imply an alternate proteoform to the one under consideration. Naturally, this is a critical consideration in using Proteomics to understand molecular/biological mechanisms as “protein machines” tend to be localized and function based on specific PTM or associated modifications or isoforms (*i.e.*, proteoforms), and stoichiometry is critical to physiologically functional molecular interactions and reactions.

### 4.4. Additional Limitations

As a final consideration of Figure 1 and Figure 2, with regard to Quadrants A and C, there may be circumstances in which neither approach (*i.e.*, Top-down or Bottom-up) is viable, including very low abundance proteins transiently present in signaling cascades. Transcriptome analyses may be necessary to at least *infer* the presence of a given protein. As mRNA analysis provides only a surrogate assessment of the actual proteins (and notably not of proteoforms *per se*), this can only be regarded as a qualitative analysis and any findings require parallel validation by PCR (*i.e.*, to address variability in microarray analyses) as well as orthogonal validation at the protein level if at all possible (e.g., high sensitivity Western blotting [36]). The tendency toward lack of criteria to further refine the “hit-list” is however a major complication of transcriptomic analyses and this is also a level of validation that warrants further critical consideration for this approach as a whole, but also particularly in terms of seeking to align these data with those obtained from other ‘omic analyses [37]. However, such gene expression analyses also highlight the temporal limitations of current Proteomic strategies, in particular for Discovery Proteomics. It is likely easier to analyze multiple samples across the time domain using the techniques of Targeted Proteomics, including protein and antibody arrays. Naturally, the quality of both the targets and the corresponding analytical reagents used in such analyses define the ultimate usefulness of the results in any diagnostic or comparable application.

## 5. Summary and Conclusions

We have shown that fitness-for-purpose is a function not only of methodology but also of sample type and we have discussed this for what we consider to be the three major objectives in protein characterization. There is *never* an acceptable reason to suspend the well-established standards of Analytical Chemistry with regard to biological and technical replicates, although the cost of some analyses is often used as a rationale. One is forced to question the statistical integrity of results produced without suitable replicate analyses even if one can sympathize with the cost issues, and also whether fitness-for-purpose was considered in designing the analyses. Furthermore, one type of replicate does not “trump” the other; analyses must include sufficient biological replicates as well as (minimally) triplicate analyses of each of these. *As soon as one moves from the intact native system to the resolution and analysis of macromolecules, the established rules of Analytical Chemistry hold sway*. Quality of analyses is paramount and far exceeds the importance of throughput; the latter has thus not been considered as a central criterion in this discussion. In apparent contrast to much of current thinking, large volumes of data are not the automatic hallmark of a good analysis; rather it is the genuine *quality* of the data from which effective lessons are learned, biological insights are made, and true knowledge arises. Simply generating lists of “potential” proteins is not consistent with the principles of good Analytical Chemistry. Thus, for the dissection of molecular mechanisms or looking for subtle differences between protein species that may well underlie disease/dysfunctional states, one would have to first consider the use of high resolution Top-down analyses (e.g., 2DE) that routinely capture those differences, in multiple samples, resolved in parallel, in multiple gels. Certainly such molecular differences can also often be resolved using shotgun strategies, but require standard triplicate analyses for each particular modification in question (e.g., *fully replicated* analytical runs for phosphorylation, oxidation, glycosylation, prenylation, nitrosylation, protonation, and so forth). At best, this would seem impractical if not completely prohibitive, if only in terms of instrument time required for the many multiple analytical runs for each aliquot (at least in triplicate) from each of at least triplicate biological replicates in an experiment. Suspending the established standards of Analytical Chemistry (e.g., ignoring multiple technical replicates of each of multiple experimental samples) seems to be the most common way of dealing with these issues of cost, instrument time, and throughput, but at the price of sacrificing analytical rigor and quality. Furthermore, it is at best questionable if a truly quantitative analysis can be done following selective prefractionation due to the high probability of a broad-spectrum loss of uncharacterized percentages of many protein species bound, for instance, to the high abundance proteins targeted for removal. At best, any such approach must also include a detailed analysis of the removed fraction in an effort to at least account for the full spectrum of species lost. Nonetheless, any sort of meaningful quantitative analysis seems unlikely if not impossible particularly as fractionation may well also bias the representation of species still remaining in the analyte mixture. Regrettably, such detailed analysis seems almost never to be done. In this regard, we think it noteworthy that simplifying a problem does not make the problem simple—and certainly not from the perspective of quantitative analyses of proteoforms.

Considering the (i) current status of Proteomics as a discipline; (ii) breadth of analytical techniques, strategies, and instrumentation; and (iii) goals of the analyses and issues of variance and reproducibility, it is unfortunate that there appears to be a trend toward instrumentation-centric thinking in some approaches rather than more rigorous addressing of the breath of criteria that must be considered in order to best ensure the excellence of analyses. Unfortunately, some organizations and journals said to represent the broader discipline of Proteomics have come to support if not define this trend. We would suggest that there is too much emphasis on Bottom-up approaches and, as a consequence, less rigorous consideration of overall data quality and capacity to translate that to genuine understanding of molecular mechanisms. There is thus considerably less attention directed toward the ambiguities in assigning identity to a protein, let alone a particular proteoform. Nonetheless, when the rigorous standards of Analytical Chemistry are observed in both types of analyses, then Top-down and Bottom-up approaches can perhaps best be considered as complementary to one another.

Thus, while instrument/technique development, adaptation and integration are obviously necessary and important, so is the parallel development, refinement and application of the best possible available techniques, tools and strategies to addressing current critical issues in healthcare, agriculture/animal husbandry, and the environment. *The well-established standards of Analytical Chemistry must apply*. It is thus up to the individual investigator to recognize the potential limitations of any data arising from the measurements made using a particular approach or combination of techniques and instruments. However, here, we have considered only the ideal situations; readers will have to determine for themselves how to justify the fitness-for-purpose of the technique(s) they choose to apply to each separate biological problem they investigate.
